# Trends in Faecal Zonulin Concentrations in Paediatric Patients with Celiac Disease at Baseline and on a Gluten-Free Diet: Exploring Correlations with Other Faecal Biomarkers

**DOI:** 10.3390/nu16050684

**Published:** 2024-02-28

**Authors:** Miguel Ángel Martínez Gallego, María Gema Crespo Sánchez, María Gemma Serrano Olmedo, Antonio Buño Soto, Sonia Álvarez Casasempere, Pilar Nozal, Eva Martínez-Ojinaga, Manuel Molina Arias, Itsaso Losantos-García, Marta Molero-Luis

**Affiliations:** 1 Department of Laboratory Medicine, La Paz University Hospital, 28046 Madrid, Spain; mmartinezgallego@salud.madrid.org (M.Á.M.G.); mariagema.crespo@salud.madrid.org (M.G.C.S.); mariagemma.serrano@salud.madrid.org (M.G.S.O.); antonio.buno@salud.madrid.org (A.B.S.); sacasasempere@salud.madrid.org (S.Á.C.); 2 Department of Immunology, La Paz University Hospital, 28046 Madrid, Spain; pilar.nozal@salud.madrid.org; 3 Centre for Biomedical Network Research on Rare Diseases (CIBERER U754), 28046 Madrid, Spain; 4 Complement Research Group, Hospital La Paz Institute for Health Research (IdiPAZ), 28046 Madrid, Spain; 5 Paediatric Gastroenterology and Nutrition Service, La Paz University Hospital, 28046 Madrid, Spain; eva.martinezojinaga@salud.madrid.org (E.M.-O.); manuel.molina@salud.madrid.org (M.M.A.); 6 Department of Biostatistics, La Paz University Hospital, 28046 Madrid, Spain; itsaso.losantos@salud.madrid.org

**Keywords:** zonulin, paediatric, celiac disease, tight junctions, intestinal permeability, calprotectin, gluten immunogenic peptides

## Abstract

Celiac disease (CeD) is an autoimmune condition triggered by gluten in genetically predisposed individuals, affecting all ages. Intestinal permeability (IP) is crucial in the pathogenesis of CeD and it is primarily governed by tight junctions (TJs) that uphold the intestinal barrier’s integrity. The protein zonulin plays a critical role in modulating the permeability of TJs having emerged as a potential non-invasive biomarker to study IP. The importance of this study lies in providing evidence for the usefulness of a non-invasive tool in the study of IP both at baseline and in the follow-up of paediatric patients with CeD. In this single-centre prospective observational study, we explored the correlation between faecal zonulin levels and others faecal and serum biomarkers for monitoring IP in CeD within the paediatric population. We also aimed to establish reference values for faecal zonulin in the paediatric population. We found that faecal zonulin and calprotectin values are higher at the onset of CeD compared with the control population. Specifically, the zonulin levels were 347.5 ng/mL as opposed to 177.7 ng/mL in the control population (*p* = 0.001), while calprotectin levels were 29.8 μg/g stool compared to 13.9 μg/g stool (*p* = 0.029). As the duration without gluten consumption increased, a significant reduction in faecal zonulin levels was observed in patients with CeD (348.5 ng/mL vs. 157.1 ng/mL; *p* = 0.002), along with a decrease in the prevalence of patients with vitamin D insufficiency (88.9% vs. 77.8%). We conclude that faecal zonulin concentrations were higher in the patients with active CeD compared with healthy individuals or those following a gluten-free diet (GFD). The significant decrease in their values over the duration of the GFD suggests the potential use of zonulin as an additional tool in monitoring adherence to a GFD.

## 1. Introduction

Celiac disease (CeD) is a chronic enteropathy triggered by an autoimmune response to gluten that occurs in genetically predisposed individuals, which can manifest at any stage of life, spanning from early childhood to advanced age [1]. The global prevalence of CeD is estimated to be 1.4% by serology and 0.7% by biopsy worldwide, mainly in the Caucasian population [2].

The atrophy of intestinal villi in CeD leads to the malabsorption and maldigestion of nutrients, resulting in symptoms such as diarrhoea, abdominal pain, weight loss, and steatorrhea. The intestinal form of CeD is more commonly detected in the paediatric population and in children younger than 3 years upon introducing gluten-containing cereals into the diet [3], whereas in older children and adults, extraintestinal signs are more frequent [4]. Malabsorption syndrome with chronic diarrhoea, weight loss, and significant asthenia is rare in adults [5].

Currently, the only treatment for CeD is based on the complete elimination of gluten from the diet for life, which implies the complete exclusion of those foods and meals, produced from wheat, rye, barley and some varieties of oats. Following the GFD can reverse intestinal damage, reduce inflammation and antibody levels, improve symptoms and prevent the development of complications associated with gluten consumption [1]. Following a GFD is far from straightforward, mainly due to gluten cross-contamination, as gluten is not only found in wheat-based foods, but also as a thickener in sauces or as a stabilising or flavouring additive. Furthermore, a GFD must not only be gluten-free, but also balanced, covering all energy and nutrient needs [6]. According to the “Codex Alimentarius Standard for Gluten-Free Foods” (Codex-standard), dietary foods labelled as gluten-free must not exceed 20 mg of gluten per kg of food when sold to consumers [7].

Intestinal permeability (IP) plays a crucial role in the pathogenesis of CeD. The mucosa of the gastrointestinal tract acts as a highly specialised barrier that separates the internal from the external environment, essential for maintaining organismal homeostasis and preventing the passage of harmful substances and microorganisms into the circulatory system [8]. Tight junctions (TJs) are the primary structures responsible for maintaining the integrity of the intestinal barrier. Zonulin, a 47 kDa human protein, plays a vital role in modulating TJs permeability, which is fundamental for maintaining physiological processes in the intestine [9] and has emerged as a potential non-invasive biomarker to study IP. TJs fail in CeD, allowing undigested gluten peptides to pass through the epithelial barrier, triggering an immune response involving both adaptive and innate immune systems [10].

Given that zonulin is associated with a series of immune-mediated diseases, a quantitative sandwich enzyme-linked immunosorbent assay (ELISA) has been developed, employing its levels in serum and stools as a biomarker of intestinal barrier integrity in several inflammatory diseases [11]. Since it has been widely shown that zonulin is not just pre-haptoglobin, but a set of proteins related structurally and functionally [12] to each other, in this study we will refer to zonulin as zonulin-related proteins (ZRPs).

In gastrointestinal inflammatory processes, there is an increase in faecal calprotectin levels, making this biomarker highly useful for differentiating organic inflammatory processes from purely functional ones [13]. Although the biomarker’s main utility is in inflammatory bowel disease (IBD), a number of authors have reported elevated calprotectin levels at the onset of CeD, especially when there are intestinal symptoms and significant histological changes [14,15]. Moreover, it has been described that villous atrophy alters exocrine pancreatic secretion [16], detected by very low faecal elastase levels, leading to poor macronutrient digestion. Both faecal biomarkers are frequently determined at the onset of CeD.

Although it is well known that ZRP levels are elevated at the onset of CeD, there are currently no studies reporting their progression once the gluten-free diet (GFD) has been established. The aim of this project was to investigate how faecal ZRP levels progress from disease onset to the first follow-up once the GFD is established. We also investigated the relationship between faecal ZRPs and other faecal and serological biomarkers and clinical variables. Prior to proceeding to these objectives, we determined faecal ZRPs’ reference levels in the paediatric population.

## 2. Materials and Methods

### 2.1. Study Design

A single-centre prospective observational study was conducted in a cohort of 23 children aged <18 years, newly diagnosed with CeD, and following a GFD for at least 6 months. The study took place between January 2022 and April 2023 at La Paz University Hospital (Madrid, Spain).

The patients attended 2 study visits: one at the time of CeD diagnosis (CeD-Onset group) and another at least 6 months later, while still following a GFD (CeD-GFD group). Each visit included a clinical examination and blood and faecal sample collection. All 23 patients provided stool and serum samples at both visits. Faecal and blood biomarkers related to CeD and IP were analysed.

**Figure 1:**
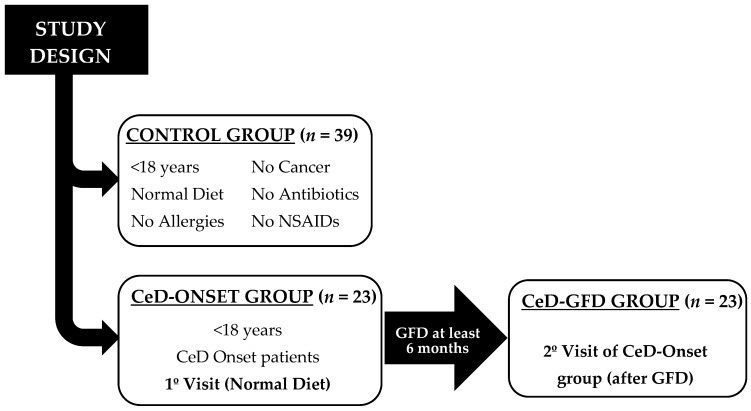
Study design. NSAID: non-steroidal anti-inflammatory drugs.

The control group (CTRL group) consisted of 39 healthy volunteer children aged <18 years old without any gastrointestinal disorders or CeD and who were following a gluten-containing diet (confirmed by positive GIPs). The exclusion criteria included any organic gastrointestinal disease, the presence of any diagnosed cancer, adherence to a GFD, self-reported allergy, regular use of non-steroidal anti-inflammatory drugs, and antibiotic use in the previous month (Figure 1). Stool samples were collected from this group to establish reference values (RVs) for faecal ZRPs and to verify paediatric calprotectin and elastase reference ranges.

### 2.2. Study Population

The diagnosis of CeD in the 23 study patients was any of the following:

- Fulfilling the European Society for Paediatric Gastroenterology Hepatology and Nutrition (ESPGHAN) criteria [17]: Children with elevated anti-tissue transglutaminase (TTG)-IgA values (10-fold or more the upper limit of normal −10 × ULN−) and positive endomysial antibodies (EMA-IgA) in a second serum sample;
- Biopsy approach: children with TTG-IgA but lower titres (<10 × ULN) and duodenal atrophy suggestive of CeD (Marsh II or III). In Marsh I findings, an intraepithelial lymphogram by flow cytometry was performed.

The inclusion criteria for the CeD-Onset group were to be diagnosed at the onset of CeD, be younger than 18 years of age, and have a written informed consent for the study by their parents. Exclusion criteria included patients already diagnosed with CeD, failure to submit a faecal sample, or failure to sign the informed consent document.

### 2.3. Ethical Considerations

All patients agreed to participate in the study and signed their written informed consent. The research was conducted in accordance with the Declaration of Helsinki and with approval from the Ethics Committee for Clinical Research at La Paz University Hospital (approval number: PI-5528).

### 2.4. Faecal Zonulin-Related Proteins (ZRPs) Analysis Validation: Assay Variables

To study the intra-individual variations, faecal ZRPs were analysed in 2 healthy volunteers (3 and 5 years of age, respectively) for 5 consecutive days.

To evaluate assay imprecision, we calculated the within-run and between-run coefficients of variation (CV = standard deviation/mean faecal ZRPs values × 100) in 5 replicates.

After these metrological validation studies, RVs were established in faecal samples.

### 2.5. Faecal Samples

The patients provided a faecal sample collected at home using a standard sample container provided by the laboratory. Upon arrival at the clinical laboratory, the samples were immediately frozen and stored at −20 °C. The samples were processed within 3 months for ZRPs, within 10 days for calprotectin and elastase, and on the same day to study immediate principles in the stool.

Faecal ZRPs were analysed in duplicate using an ELISA kit (Zonulin Stool ELISA, DRG Instruments Gmbh, Marburg, Germany), and the optical density was measured with a photometer (DYNEX DSX System, Dynex Technologies, Chantilly, VA, USA) at 450 nm and 620 nm. Duplicates’ coefficients of variation (CVs) were calculated, and the results were expressed as ng/mL.

Faecal calprotectin and elastase were extracted using the Calprotectin Stool Extraction Device (DiaSorin S.p.A., ref X0043, Saluggia, Italy) and analysed on the LIAISON^®^XL analyser following the manufacturer’s protocol (DiaSorin S.p.A., ref 318960). The calprotectin concentration in the sample was determined on the standard curve and expressed in micrograms of calprotectin per gram of stool (μg C/g stool, which is equal to mg C/kg stool). Reference values of 50 μg/g and >200 μg/g were taken as normal for calprotectin and elastase, respectively, according to the manufacturer’s recommendations.

Faecal gluten immunogenic peptides (GIP) were assessed for several purposes: in the control group to verify that they were consuming gluten; in the CeD patients at the time of debut to ensure that patients were still consuming gluten; and at disease follow-up to assess adherence to the GFD. Stool GIP concentrations were determined by a sandwich ELISA (iVYDAL In Vitro Diagnostics iVYLISA GIP Stool Kit, Biomedal S.L., Seville, Spain) according to the manufacturer’s protocol. The results are therefore expressed in μg GIP per gram of faeces.

To study maldigestion and the malabsorption of macronutrients in faeces, the process included staining a stool sample with Lugol’s iodine solution to detect undigested starch granules and muscle fibres. Sudan stain was also applied to identify fat globules. The preliminary screening involved placing the stool on a slide, staining it with Lugol and Sudan, and observing it under an optical microscope at 20× and 40× magnification. This served as an initial screening test for maldigestion and malabsorption, assisting physicians in orienting themselves to these disorders.

### 2.6. Serum Samples

Blood samples were collected to obtain serum and were stored at −80 °C until analysis. All serum samples were tested for TTG-IgA and anti-endomysial antibody IgA (EMA-IgA). TTG-IgA levels were measured by a fully automated fluoroenzyme immunoassay, using the ImmunoCAP EliA Celikey system (Thermo Fisher Scientific, Uppsala, Sweden), according to the manufacturer’s protocol. The results are expressed as UI/L. The manufacturer recommends a cut-off of >10 UI/L as a positive test. Immunofluorescent analysis of EMA-IgA was performed by an experienced immunologist, using serum dilutions of 1:5.

### 2.7. Statistical Analysis

The distribution of variables in the population was assessed using the Kolmogorov–Smirnov test. Continuous variables conforming to a normal distribution were described using the mean and standard deviation, whereas those deviating from a normal distribution were characterised using the median and interquartile range. Categorical variables are presented in terms of absolute frequencies and percentages.

For the comparison of categorical variables between the control group and the CeD and GFD groups, Pearson’s chi-squared test was applied. Continuous variables with a Gaussian distribution were compared using Student’s *t*-test, whereas those not adhering to a Gaussian distribution were assessed with the Mann–Whitney U test.

For the paired data analysis, a paired Student’s *t*-test was employed for variables following a normal distribution at both time points, whereas Wilcoxon’s signed-rank test was employed for variables not demonstrating normality at both time points.

To explore potential correlations between parametric variables, Pearson’s correlation coefficient was employed, while Spearman’s Rho was used for non-parametric variables.

Data processing was conducted using the statistical programming language R (version 4.2.1, R Core Team, 2020). Each test was 2-tailed, with a 95% confidence level.

## 3. Results

### 3.1. Assay Variables

The intra-individual variation between the two volunteers showed a CV value of 33.2% and 29.2%, respectively.

Regarding the imprecision assay studies, the within-run CV was 28.5% for faecal ZRPs of 392.5 ng/mL and the between-run CV was 14.2% for faecal ZRPs of 219.0 ng/mL. Faecal ZRPs were analysed in duplicate. Of the studied samples, 90% showed a variation in duplicates of <10%, 5% exhibited a variation of 10–15%, and the remaining 5% displayed a variation of >15%.

### 3.2. Establishment of Reference Values

In the present study, faecal samples from 39 healthy paediatric individuals (CTRL group) (mean age 7.2 ± 4.5 years; 71.4% female) were analysed. RVs for faecal ZRPs were established according to age. Given that there was no statistically significant relationship between ZRPs and age, a single reference interval was defined. Table 1 shows the descriptive parameters for the faecal ZRPs, as well as for calprotectin and elastase, in the control group.

Furthermore, faecal and blood serum samples from the 23 paediatric patients in the CeD-Onset group (mean age, 7.5 ± 3.6 years; 65.2% female) were analysed. These patients were subjected to a GFD for a mean of 6.8 ± 2.2 months from diagnosis (CeD-GFD group).

### 3.3. Patients with Newly Diagnosed Celiac Disease

#### 3.3.1. Genetic and Histological Study

Genetic testing for HLA-DQ2 and HLA-DQ8 was conducted on 20 of the 23 patients in the CeD group to determine the HLA-associated CeD risk and to complete the patient study. Among them, 11 were identified with a very high risk, and 9 exhibited a high risk.

In 5 of the 23 patients, a duodenal biopsy was performed to establish a CeD diagnosis. Of the four patients with positive biopsy results, three had Marsh Stage IIIb (partial villous atrophy) and one exhibited Marsh Stage IIIc (total villous atrophy). The patient who did not present intestinal damage (Marsh Stage I, increased lymphocytes in the duodenal epithelium) was diagnosed with CeD based on a distinctive lymphogram profile, showing elevated gamma-delta lymphocytes (23.6%, normal values (VN) < 8.5%) and decreased NK cells (1.6%, VN > 10%). In the remaining 18 patients, CeD diagnosis was established by meeting the ESPGHAN criteria (TTG-IgA levels > 10 × ULN) and therefore did not undergo duodenal biopsy.

#### 3.3.2. Clinical Presentation

Of the 23 evaluated patients, 52% (12/23) experienced digestive symptoms during the onset of CeD, such as diarrhoea, abdominal distension, abdominal pain, and/or constipation. However, no significant correlation (*p* = 0.545) was found between the presence of these symptoms and faecal ZRP levels in these patients. The mean faecal ZRP value obtained in the patients with CeD and digestive symptoms was 375.3 ± 219.2 ng/mL, whereas those without symptoms had a mean value of 319.2 ± 218.1 ng/mL. No significant differences were observed between calprotectin values and digestive symptomatology.

The remaining 11 (48%) patients manifested various clinical presentations. Four of them showed a weight delay, one presented asthenia, two experienced alopecia, two had type one diabetes mellitus (T1DM), one was under follow-up for autoimmune thrombocytopenia, and one patient was under follow-up after a mononucleosis infection.

#### 3.3.3. ZRPs and Other Faecal Biomarkers

**Figure 2:**
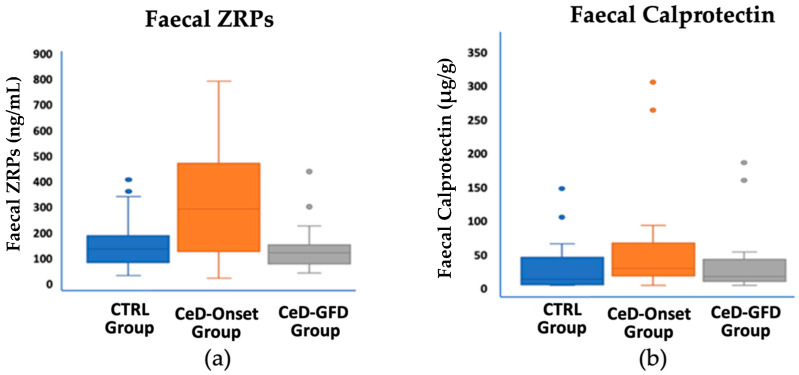
Faecal ZRPs and calprotectin values in the three study groups. (**a**) For faecal ZRPs, statistically significant differences were observed between the CeD-Onset group and the control group (*p* = 0.001) and between the CeD-Onset and CeD-GFD groups (*p* = 0.002). (**b**) For calprotectin, statistically significant differences were found between the control and CeD-Onset groups (*p* = 0.029).

Statistically significant differences were found in faecal ZRPs (t-value, −3.63; *p* = 0.001) and calprotectin (U = 298.5; *p* = 0.029) levels between the control and CeD groups (Figure 2 and Table 1). Although there were no statistically significant differences between the control and CeD groups in terms of faecal elastase, two patients were identified with very low elastase values (16.5 and 15.3 μg/g, respectively), the latter being a patient with T1DM.

In 21 of the 23 patients with newly diagnosed CeD, GIP testing was positive, because they were still consuming gluten. As for the two patients with negative GIP results, the responsible guardians reported gluten removal from their diet a few days before the faecal sample was collected. No significant differences were observed between faecal ZRP values and faecal GIPs.

#### 3.3.4. Serum Biomarkers

The median TTG-IgA value for the 23 patients was 210 U/L (IQR, 75–460 U/L). There was no statistically significant association between TTG-IgA levels and faecal ZRP levels (*p* = 0.517).

As for the other biochemical parameters analysed in serum (blood count, zinc, alanine aminotransferase [ALT], iron, transferrin, iron transport capacity, transferrin saturation index [TSI], ferritin, vitamin D, vitamin B_12_, calcium, phosphate, creatinine, glucose, cholesterol), 39% of the patients had a reduced TSI, 26.1% had elevated ALT levels (>35 U/L), and 88.9% had a vitamin D insufficiency (<30 ng/mL), a finding that was statistically significant (*p* < 0.0035; r = −0.595). In the CeD group, an inversely proportional relationship was observed between faecal ZRPs and vitamin D levels (*p* = 0.001, Pearson’s Rho = −0.64).

### 3.4. Patients under Follow-Up with GFD

#### 3.4.1. Clinical Presentation

All patients experienced an improvement in their digestive symptoms. All of the patients with delayed growth reached normal weight and height percentiles for their age.

#### 3.4.2. Faecal ZRPs and Other Faecal Biomarkers

**Figure 3:**
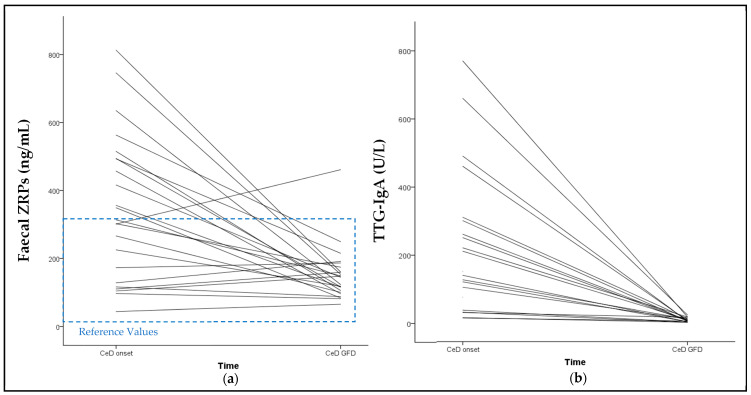
Trajectory of the reduction in faecal ZRPs and TTG-IgA overtime. (**a**) Reduction in faecal ZRPs of each CeD patient from baseline to follow-up visit. The median percentage reduction was 54.1%. (**b**) Reduction in serum TTG-IgA of each CeD patient from baseline to follow-up visit. The median percentage reduction was 96.6%. No relationship was observed between ZRPs reduction and TTG-IgA reduction. Only one patient showed an increase in ZRP levels above the RVs over time, and tested negative for GIP and TTG-IgA in the follow-up visit. The trajectory of one patient’s TTG-IgA reduction from 5800 to 18 U/L has been excluded from the graph. The ZRP reference values are represented in blue dashed lines.

All 23 patients presented a reduction in faecal ZRP values after following a GFD for 6.8 ± 2.2 months. The median percentage decrease in ZRP levels was 54.1% (from 348.5 ± 215.6 to 157.1 ± 88.9 ng/mL). Additionally, this reduction became more pronounced as the GFD duration increased (*p* = 0.010; Spearman’s Rho = 0.524). Figure 3 shows the trajectory of the reduction in ZRP values over time.

The CeD-GFD group exhibited significantly lower ZRP values than the CeD-Onset group (*p* = 0.002). However, when comparing these values with those of the control group, no statistically significant differences were observed (U = 366; *p* = 0.229) (Figure 2, Table 1). There were no statistically significant differences in calprotectin values among the GFD group when compared with the control and CeD-Onset groups.

Regarding the patients with very low elastase values, the patient with faecal elastase values at the onset of 16.5 µg/g showed an increase in elastase levels to 45.5 µg/g after 6 months of follow-up but did not reach pancreatic sufficiency. In the same stool sample, the quantification of faecal chymotrypsin (54 U/g stool, reference range >15.3 U/g) and the daily fat excretion in stools (0.9 g/day; reference range, 0.5–6.0) were non-pathological. The other patient, who had a starting elastase value of 15.3 μg/g at the onset of CeD and had T1DM, experienced an increase to 393 µg/g at 6 months on a GFD and showed positive TTG-IgA antibodies (105 U/L).

During the analysis of staining of the stool sample with Lugol’s and Sudan’s solutions, notable findings included a discernible trend towards the normalisation of muscle fibre digestion in the CeD group compared with the GFD group (57.1% vs. 76.2%). Moreover, a reduction in the percentage of patients exhibiting elevated faecal fat levels was evident in the GFD group (52.4%) in contrast to the CeD group (76.2%), reflecting a reduction of 31.2%. Additionally, a decrease in the presence of starch granules and cellulose was observed in the GFD group compared with the CeD group, with reductions of 16.7% and 7.0%, respectively. There were no differences between the CeD and GFD groups in the other parameters evaluated with the faecal digestion test, such as odour, colour, consistency, and presence of blood or mucus (Table 2).

The GIP was negative in all patients except for two patients in whom their TTG-IgA antibodies were 17.0 and 11.0 U/L, respectively. The faecal ZRP reductions in these two patients were 42.4 and 15.5%, respectively. The parents of these patients have been informed to review the GFD.

#### 3.4.3. Serum Biomarkers

The TTG-IgA analysis was conducted in 20 of the 23 patients, due to the lack of serum samples for the remaining three patients. All patients showed a decrease in TTG-IgA concentrations (Figure 3). No correlation was found between TTG-IgA reduction and ZRP reduction. Among the 20 patients tested, 12 of the 20 patients exhibited a normalisation of TTG-IgA levels (<10 U/L) with a mean GFD duration of 6.79 months. All of these patients also had a negative GIP and the median ZRP reduction was 70.7%. On the other hand, the eight patients who did not achieve negative TTG-IgA values showed a range of 11–23 U/L, with a mean GFD duration of 7.1 months. Faecal samples were only available from six patients. The median ZRP reduction among these six patients was 29.0%. No statistically significant difference was found between the ZRP reduction in TTG-IgA-positive patients compared to TTG-IgA-negative patients.

Of these six patients, two were GIP-positive and four GIP-negative, two of whom were EMA-IgA-negative and two of whom were weakly EMA-IgA-positive. The median ZRP reduction for the two GIP-positive patients was 29.0%, while for the four GIP-negative patients it was 7.3%, with no statistically significant difference between the two groups.

Regarding the patients who had abnormal biochemical results at the onset of CeD, the recovery of IST and ALT was observed in most patients but not in vitamin D levels, given that the percentage of patients with insufficiency (<30 ng/mL) changed from 88.9% to 77.8%. The mean vitamin D level in the CeD group was 23.9 ± 6.5 ng/mL versus 27.7 ± 8.2 ng/mL in the GFD group, showing no statistically significant differences.

## 4. Discussion

As far as we know, this study constitutes the first work that establishes RVs for faecal ZRPs in the paediatric population up to 16 years of age. Faecal ZRP values were determined in children aged 2 months to 16 years, obtaining a single range due to the lack of differences related to age or sex. The mean faecal ZRP value in our study was 177.7 ± 87.3 ng/mL, slightly lower than the values reported by Łoniewska [18], who conducted a single study analysing faecal calprotectin and ZRPs in healthy individuals during the first 2 years of life and observed an increase in faecal ZRP levels from birth to 2 years, with median faecal ZRP concentrations of 223.7 ng/mL and 256.9 ng/mL at 12 and 24 months, respectively. Other studies performed in adult control populations have reported values of 30 ng/mL [19].

After birth, humans’ IP to macromolecules is very high [20]. Over time, this permeability gradually decreases, a process known as “intestinal closure” [21,22], which is associated with the increased thickness and density of the intestinal mucus lining, reducing the absorption of lactoglobulins and macromolecules [23]. This phenomenon could explain the lower value of faecal ZRPs in the paediatric population of the present study.

Other studies have been conducted on the importance of ZRPs as a biomarker of IP in the paediatric population, such as the one by Tarko et al. [24], who investigated children from the second to the eleventh day of life, and the one by Saleem et al. [25], on premature infants (born before the 28th week of pregnancy). In both studies, however, serum ZRP levels were determined rather than faecal ZRP levels. It is worth noting that serum ZRPs was also quantified in our study (data on request). However, the inability to establish RVs in the same control group, the lack of correlation between faecal and serum ZRPs in CeD patients at baseline, the lack of correlation with adherence to GFD, and budgetary constraints led us to make the decision to focus only on faecal ZRPs.

The statistically significant differences in faecal ZRP values between the CeD and control groups highlight the marked IP observed at the onset of CeD, aligning with the documented literature [26,27]. Additionally, the substantial reduction (exceeding 50%) in faecal ZRP values after 6 months on a GFD emphasises the reversible nature of ZRPs and IP, as indicated by Fasano et al. [28]. Notably, we observed that this reduction becomes more pronounced with prolonged adherence to the diet, supporting the aforementioned observation. However, the absence of a statistically significant difference between the control and CeD-GFD groups suggests that, although gluten does elevate faecal ZRP levels (as observed in the non-negligible ZRP values in the control group), these levels are not elevated enough to be considered pathological and that other mechanisms are involved in causing an increase in IP and, consequently, ZRPs levels. Therefore, the adoption of a GFD without a specific medical need to reduce IP may have more risks than benefits. Our study showed that IP did not show statistically significant differences between the control group (gluten consumers) and CeD on a GFD. Furthermore, following a strict GFD is not only difficult due to its presence in many foods and condiments, but it can also lead to nutritional imbalances. Several studies have documented nutritional deficiencies, including low fibre intake [29], increased intakes of refined sugars and saturated fats due to the consumption of processed gluten-free products [30], and lower intakes of micronutrients, particularly vitamin D, vitamin B_12_, and folate, and some minerals such as iron, zinc, magnesium, and calcium [29].

Faecal calprotectin is a highly valuable diagnostic biomarker and predictor of relapse in IBD [13]. However, its effectiveness as a biomarker of IP is more limited [31]. In our study involving paediatric patients with CeD, we found no correlation between faecal ZRPs and calprotectin. A number of authors, such as Szymanska et al. [9], have reported a correlation between ZRPs and calprotectin in patients with IBD. The lack of such a relationship in our CeD population could be attributed to different pathophysiology, such as the lower number of neutrophils in the lamina propria of patients with CeD compared with those with IBD.

We observed elevated calprotectin values in patients with newly diagnosed CeD compared with the control population. Similar findings have been reported by other groups [14,15], highlighting the utility of calprotectin as a valuable biomarker at the onset of CeD, particularly in patients with gastrointestinal presentations [14] and for monitoring dietary adherence [15].

Surprisingly, we identified two newly diagnosed patients with CeD who had notably low faecal elastase values and increased fat elimination in the digestive stool test. In the follow-up, one of these patients still exhibited pancreatic insufficiency values. However, the biochemical profiles of other markers, such as chymotrypsin and indicators of malabsorption, remain within normal ranges. Despite the ongoing clinical digestive improvement in this patient, they are being closely monitored without Kreon^®^ enzyme supplementation.

In the other patient, who also had T1DM, elastase levels normalised after 6 months of a GFD. The finding of low elastase levels coincided with an episode of gastroenteritis, which by a dilutional effect could be the explanation for the low values.

No correlation was identified between ZRPs and GIP values, both at the onset and after 6 months on a GFD. In this study, we observed that CeD patients with a GFD who tested positive for GIPs and TTG-IgA had a ZRP reduction. This might suggest that in occasional transgressions (GIP-positive), there would not be sufficient release of ZRPs from intestinal epithelial cells to be quantified in faeces. This observation is consistent with the findings of the study by Drabińska [32]. However, other studies have reported the effect of gliadin in activating ZRPs’ release and increasing IP [33].

We have noticed a trend indicating improved fat digestion with the implementation of a GFD. The study of immediate principles in stool serves as a convenient and cost-effective initial screening tool, enabling us to assess macronutrient digestion more comprehensively. Leveraging our laboratory’s extensive experience with this test, we sought to investigate whether paediatric patients with CeD exhibit enhanced macronutrient digestion upon adopting a GFD.

The low vitamin D levels observed in this study (88.9%) roughly align with those in the literature (0–70%) [34]. Although the percentage of patients with vitamin D insufficiency (<30 ng/mL) decreased in our cohort, we still consider it relatively high (77.8%). Following the recommendations in the literature, we emphasise the importance of closely monitoring the nutritional vitamin D status of patients with CeD. Additionally, it is important to note that vitamin D levels tend to be particularly low in the general population [34].

The disappearance of detectable GIP in the celiac follow-up population, combined with the normalisation of TTG-IgA in most patients, underscores the importance of assessing serum and faecal markers in CeD monitoring.

A significant issue that could impact ZRPs’ measurements is the method employed by commercial ELISAs for ZRPs’ quantification. Several publications have reported that these ELISAs simultaneously detect various proteins structurally and functionally resembling ZRPs [18,35]. In certain situations, the ZRP value is underestimated. One explanation is that the tertiary and quaternary structural conformation adopted by ZRPs in samples differs from what ELISAs can detect [12]. For this reason, our study preferentially referred to them as ZRPs rather than solely zonulin.

We would like to point out certain limitations in our study. First, we acknowledge that our study employs an exploratory approach to the progression of ZRPs during the debut and follow-up of CeD in the paediatric population, given that the number of individuals in the experimental groups is small. Second, we did not analyse the standard biomarker for assessing IP (urinary lactulose-mannitol), mainly because we did not want to change the established timeline of newly diagnosed paediatric patients with CeD at our hospital. Patients were only asked to provide a stool sample on the day of the second analysis (for those meeting ESPGHAN criteria) or on the day of biopsy. Third, the group chosen to define the RVs should be expanded to ensure the established values. Despite these limitations, to the best of our knowledge, our study is the first to estimate faecal ZRP reference values in the paediatric population up to 16 years of age, which we believe could contribute to the scientific literature.

## 5. Conclusions

In our exploratory study, we have been able to establish RVs of faecal ZRPs in our paediatric population. Furthermore, faecal ZRP concentrations were higher in the paediatric patients with active CeD than in the control and CeD-GFD groups. The significant decrease in their values over the duration of the GFD suggests the potential use of ZRPs as an additional tool in monitoring adherence to a GFD, as well as evaluating a decrease in IP observed in these patients once the adverse effect of gluten is eradicated. However, larger and more validated studies are needed to use ZRPs as a marker of IP and to monitor adherence to a GFD.

## Figures and Tables

**Table 1:** Descriptive statistics of faecal ZRPs, calprotectin, and elastase in the faeces of the three groups in the study.

		Faecal ZRPs (ng/mL)				Faecal Calprotectin (µg/g Stool)				Faecal Elastase (µg/g Stool)			
	*n*	Mean (SD)	Median (IQR)	Min–Max	RV	Mean (SD)	Median (IQR)	Min–Max	RV	Mean (SD)	Median (IQR)	Min–Max	RV
CTRL group	39	177.7 (87.3)	158.5 (103.5)	56.0–429.0	3–352	49.0 (143.2)	13.9 (39.5)	5.0–148.0	≤87	793.3 (29.2)	800.0 *	800 *	>200
CeD-Onset group	23	348.5 (215.6)	313.5 (366)	44.0–813.0	-	58.0 (76.6)	29.8 (48.8)	5.0–306.0	-	661.8 (257.1)	800 (345.0)	15.3–800	-
CeD-GFD group	23	157.1 (88.9)	144.5 (74)	65.0–461.0	-	53.7 (92.1)	17.4 (33.5)	5.0–421.0	-	651.1 (260.1)	800 (754.5)	45.5–800	-

SD: standard deviation; IQR: interquartile range; Min–Max: minimum and maximum values. * The faecal elastase samples were not diluted, with the quantification limit of the technique set at 800 µg/g.

**Table 2:** Changes in the presence of macronutrients in faecal samples (%) between the CeD-Onset group and CeD-GFD group.

	Observed Frequencies (%)			
	Increased Starch Granules	Increased Fat	Increased Cellulose	Digested Muscle Fibres
CeD-Onset group	85.7	76.2	66.7	57.1
CeD-GFD group	71.4	52.4	61.9	76.2
% Reduction ^1^	16.7	31.2	7.0	−33.4

^1^ Percentage reduction = ((CeD-Onset group − CeD-GFD group)/CeD-Onset group) × 100.

## Data Availability

The data used in this study are available from the corresponding author upon reasonable request.
